# Development of a Sensitive and Specific RPA-CRISPR/Cas12a Assay for Intrahepatic Quantification of HBV cccDNA

**DOI:** 10.3390/ijms27010551

**Published:** 2026-01-05

**Authors:** Pattida Kongsomboonchoke, Chaiyaboot Ariyachet, Pornchai Kaewsapsak, Pongserath Sirichindakul, Pisit Tangkijvanich

**Affiliations:** 1 Center of Excellence in Hepatitis and Liver Cancer, Faculty of Medicine, Chulalongkorn University, Bangkok 10330, Thailand; gee.pattida@gmail.com (P.K.);; 2 Department of Biochemistry, Faculty of Medicine, Chulalongkorn University, Bangkok 10330, Thailand; 3 Department of Surgery, Faculty of Medicine, Chulalongkorn University, Bangkok 10330, Thailand

**Keywords:** covalently closed circular DNA, recombinase polymerase amplification, CRISPR/Cas12a

## Abstract

Hepatitis B virus (HBV) persists in infected hepatocytes through covalently closed circular DNA (cccDNA), a stable episomal form that serves as the transcriptional template for viral replication. Accurate and sensitive quantification of intrahepatic cccDNA is crucial for evaluating antiviral therapies, particularly those targeting a functional cure. Here, we report the development of a novel, cccDNA-specific detection system combining recombinase polymerase amplification (RPA) with CRISPR/Cas12a-based fluorescence detection. We designed and validated CRISPR RNAs (crRNAs) targeting HBV cccDNA-specific regions conserved across genotypes A–D. Reaction conditions for both RPA and Cas12a detection were optimized to enhance sensitivity, specificity, and accuracy. The system reliably detected as few as 10 copies of cccDNA-containing plasmid per reaction and showed no cross-reactivity with non-cccDNA forms in serum or plasma, indicating assay specificity. When applied to liver tissue samples from 10 HBV-infected and 6 non-HBV patients, the RPA-CRISPR/Cas12a assay exhibited a high sensitivity (90%) and a strong correlation with qPCR results (R^2^ = 0.9155), confirming its accuracy. In the conclusion, the RPA-CRISPR/Cas12a system provides a robust, cost-effective, and scalable platform for sensitive and specific quantification of intrahepatic HBV cccDNA. This method holds promises for research and high-throughput therapeutic screening applications targeting cccDNA clearance.

## 1. Introduction

Hepatitis B virus (HBV) infection remains a significant global health challenge, with an estimated 254 million people chronically infected, according to the World Health Organization (WHO). Chronic HBV infection (CHB) contributes to substantial morbidity and mortality, with approximately 1.1 million deaths in 2022, primarily due to cirrhosis and hepatocellular carcinoma (HCC) [1]. Without novel therapeutic interventions, the global mortality rate from HBV-related diseases is projected to increase by 39% by 2030, despite the availability of safe and effective vaccines [2].

A key factor in the persistence of CHB is the covalently closed circular DNA (cccDNA), an episomal form of the viral genome that resides in the nuclei of infected hepatocytes. cccDNA acts as the transcriptional template for HBV pregenomic RNA (pgRNA) and viral proteins, sustaining viral replication. Although cccDNA lacks an origin of replication and is not propagated through semiconservative replication, its stability within quiescent hepatocytes allows it to persist and maintain chronic infection [3]. A functional cure for CHB is characterized by cccDNA elimination, loss of hepatitis B surface antigen (HBsAg), durable HBV DNA suppression, and normalization of alanine aminotransferase (ALT) levels [3,4]. However, current therapies, including nucleos(t)ide analogues (NAs) and interferons (IFNs), do not target cccDNA directly, necessitating long-term treatment. New therapeutics under development, such as terbinafine [5] and PBGENE-HBV [6], aim to suppress and eliminate cccDNA, respectively.

cccDNA often exists at extremely low copy numbers, particularly in cases of occult hepatitis B infection (OBI), where patients are HBsAg-negative but may harbor low levels of HBV DNA in liver or serum [7]. Detecting cccDNA is further complicated by its high sequence homology with relaxed circular DNA (rcDNA), necessitating highly sensitive and specific detection methods [8]. The gold standard for cccDNA detection is Southern blotting (SB), which excellently separates cccDNA from protein-free rcDNA (pf-rcDNA) based on electrophoretic mobility [9]. Despite its specificity, SB is labor-intensive, time-consuming, and exhibits limited sensitivity (limit of detection (LOD) was about 10^5^–10^6^ copies) [10], rendering it unsuitable for high-throughput settings [8]. Quantitative PCR (qPCR) offers a practical alternative, widely used in research and clinical settings. qPCR uses gap-spanning primers to amplify cccDNA selectively and the copy number is quantified relying on a calibration curve [8,11]. However, false-positive signals due to rcDNA and replicative intermediates (RIs) can lead to overestimation [8,11,12]. Enzymatic treatments (e.g., PSAD, T5 exonuclease, exonuclease I/III) have been incorporated to improve specificity [9,13,14,15,16]. Droplet digital PCR (ddPCR) enables absolute quantification with high sensitivity and specificity, but it requires costly instrumentation and specialized training [17,18].

The CRISPR/Cas system, originally discovered as an adaptive immune mechanism in bacteria [19,20,21], has recently emerged as a powerful biosensing platform for nucleic acid detection [22]. CRISPR-associated nucleases recognize specific nucleic acid targets (double-stranded DNA (Cas12), single-stranded DNA (Cas14), or RNA (Cas13)) and subsequently induce collateral cleavage of nearby single-stranded nucleic acids [23,24,25,26,27,28]. This trans-cleavage activity can be harnessed using quenched fluorophore-labeled probes that emit fluorescence upon target recognition [23,26,29]. CRISPR-based diagnostics provide high sensitivity, specificity, cost-effectiveness, and operational simplicity, making them well-suited for point-of-care testing (POCT) applications, including lateral flow and fluorescence-based assays [30,31,32,33,34,35,36,37]. Although CRISPR/Cas12a has been applied for the detection of HBV DNA in serum samples [38,39], only the Cas13a-based assay has been reported for cccDNA detection [40]. Despite achieving high sensitivity and specificity, the Cas13a method requires converting the cccDNA template into RNA and involves handling both RNA templates and RNA reporters, which increases complexity and technical difficulty in assay development.

Here, we report the development of a simplified, highly sensitive CRISPR/Cas12a-based detection system for HBV cccDNA. Cas12a directly targets double-stranded DNA (dsDNA) and cleaves single-stranded DNA (ssDNA) reporters, avoiding the need for RNA intermediates. We integrated recombinase polymerase amplification (RPA) for pre-amplification and optimized the assay for high-throughput cccDNA quantification with excellent specificity and sensitivity.

## 2. Results

### 2.1. Design and Selection of cccDNA-Specific crRNAs

**Figure 1:**
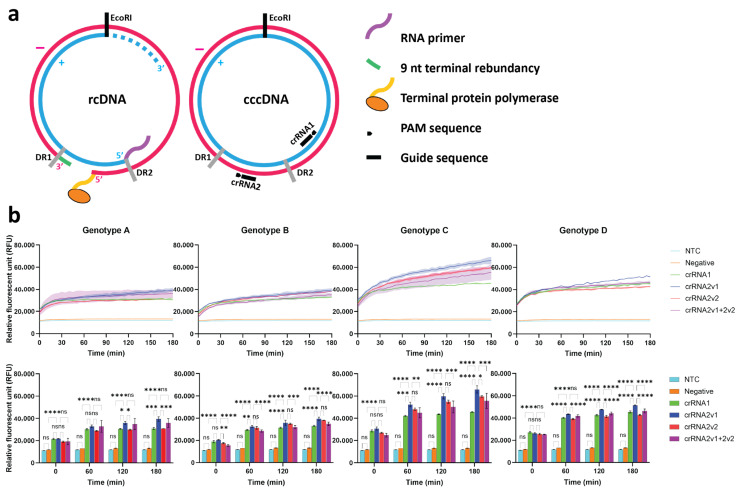
Design and selection of crRNAs. (**a**) Schematic representation of HBV rcDNA and cccDNA structures. Two DRs are located at nucleotide positions 1592 and 1826, with numbering beginning at the *Eco*RI site. crRNA1 targets the region opposite the minus strand near DR2, while crRNA2 targets the region opposite the gap in the rcDNA. (**b**) Screening of crRNAs for cccDNA detection using Cas12a collateral cleavage activity. Experiments were performed in technical triplicate. Statistical significance is indicated as follows: * *p* < 0.05; ** *p* < 0.01; *** *p* < 0.001; **** *p* < 0.0001; ns, not significant.

To distinguish HBV cccDNA from rcDNA, we targeted structurally unique regions of cccDNA, including the gap region of rcDNA and the 100 nt long opposing region of the incomplete plus strand located upstream of direct repeat 2 (DR2), which are present only in cccDNA (Figure 1a). Candidate crRNAs were designed within target regions using the CRISPOR tool [41] and aligned across HBV genotypes A–D using Jalview [42] to ensure conservation. As shown in Figure S1, two crRNAs, crRNA1 and crRNA2, were 23 nt long at the downstream next to the TTTA protospacer adjacent motif (PAM) sequence of Cas12a. They were conserved among HBV genotypes A–D. crRNA1 shows the variation at the 6th nucleotide (T = 61.8%), while crRNA2 contains variations at the 15th and 17th nucleotides next to PAM (A = 71.6% and C = 81.7%, respectively). From Table 1, crRNA2 showed higher predicted activity and was further optimized into two variants, crRNA2v1 and crRNA2v2, to match genotypes B/D and C, respectively. The crRNA variants were expected to be used in combination for comprehensive cccDNA targeting among HBV genotypes. Additionally, no off-target effects were identified in the human genome. As shown in Figure 1b (upper panels), all candidate crRNAs and a combination of crRNA2v1 and crRNA2v2 generated strong fluorescence signals in Cas12a reactions using plasmid templates across genotypes, compared with the negative control and the non-template control (NTC). Moreover, crRNA2v1 consistently yielded the highest signal intensity in kinetics detection (Figure 1b, lower panels). Thus, crRNA2v1 was selected for subsequent experiments and renamed to crRNA2.

### 2.2. Optimization of the RPA and Cas12a-Based Assays

**Figure 2:**
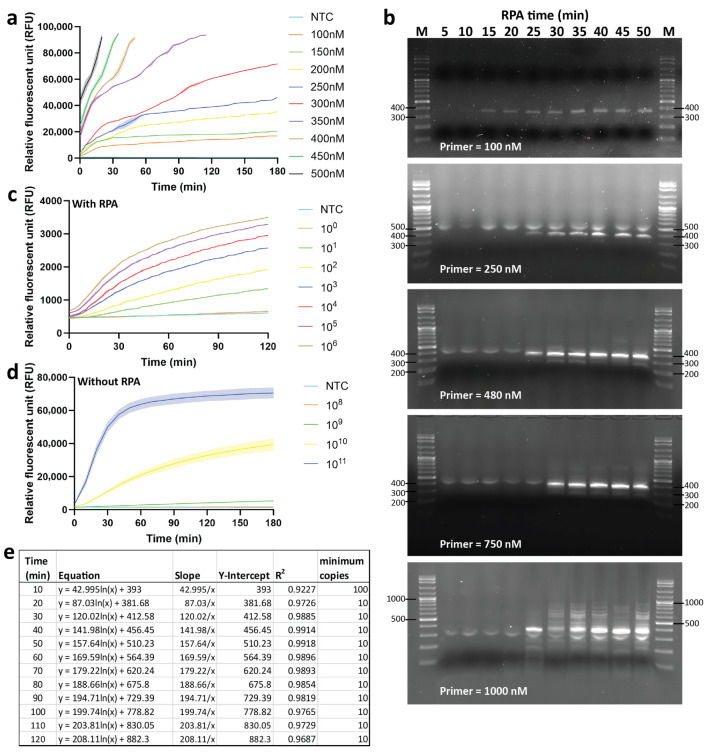
Optimization of the RPA-CRISPR/Cas12a system. (**a**) Optimization of ssDNA reporter concentration, tested at 100–500 nM. (**b**) Optimization of primer concentrations (100–1000 nM) and RPA incubation times (5–50 min). The expected cccDNA-specific amplicon is 344 bp. M = 100 bp DNA ladder. (**c**) Collateral cleavage activity of CRISPR/Cas12a in combination with RPA or (**d**) without RPA to evaluate detection sensitivity. (**e**) Linear correlation between fluorescence intensity and template copy number at various time points. The minimum detectable cccDNA copy number was defined as the lowest number with a signal above the detection limit.

We first optimized the concentration of the ssDNA reporter from 100–500 nM using a high-copy number plasmid as a template. High concentrations (≥350 nM) led to fluorescence saturation, while lower concentrations (≤150 nM) produced diminished reaction kinetics. A final concentration of 300 nM provided the most consistent fluorescence dynamics and was selected for all subsequent reactions (Figure 2a). Next, we optimized RPA conditions. cccDNA-specific primers were obtained from a previous report [17]. Only each primer at 100 nM yielded specific amplification without non-specific by-products (Figure 2b). RPA reactions performed for 50 min produced maximal yield; prolonged incubation (>70 min) resulted in non-specific amplification (Figure S2). Hence, we selected a 100 nM final concentration of primers and a 50-min incubation time for the RPA reaction.

The sensitivity of the RPA-CRISPR/Cas12a assay was evaluated using serial dilutions of cccDNA-containing plasmids. Templates were pre-amplified by optimized RPA with cccDNA-specific primers, reported previously [17], and reactions were stopped by heat-inactivation. Then, fluorescent signals were measured from the trans-cleavage activity of Cas12a. Sensitivity tests demonstrated that RPA-CRISPR/Cas12a could detect cccDNA as few as 10 copies/reaction (Figure 2c). In contrast, direct Cas12a detection without RPA could only distinguish concentrations above 10^9^ copies/reaction (Figure 2d). Regression analysis revealed a strong correlation (R^2^ > 0.9) between fluorescence intensity and template copy number at all 10-min interval time points (Figure 2e and Figure S3). Notably, the strongest correlation was presented at 50 min of reaction time, which was selected as the optimal incubation time for detection.

### 2.3. Specificity of Cas12a-Based Detection

**Figure 3:**
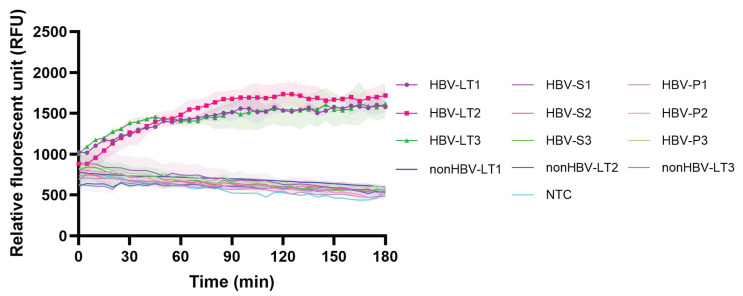
Specificity of RPA-CRISPR/Cas12a-based cccDNA detection in clinical samples. cccDNA detection was evaluated in various clinical sample types: liver tissue (LT), serum (S), and plasma (P). Samples were obtained from HBV-positive (HBV) and HBV-negative (nonHBV) individuals. NTC = non-template control.

To evaluate the assay’s specificity, we analyzed HBV-positive liver tissue, serum, and plasma from three patients with HBsAg-positive and high viral loads, alongside liver tissues from three individuals without historical records of HBV infection as negative controls (Table S1). Genomic DNA or pf-DNA was extracted from each specimen and subjected to enzymatic pre-treatment. After the RPA, fluorescent signals were generated through Cas12a-mediated collateral cleavage. The results demonstrated that cccDNA was detected exclusively in liver tissue samples, not in serum, plasma, or control liver tissue, confirming the assay’s tissue specificity (Figure 3).

### 2.4. Comparison of RPA-CRISPR/Cas12a with qPCR and ddPCR for cccDNA Quantification

**Figure 4:**
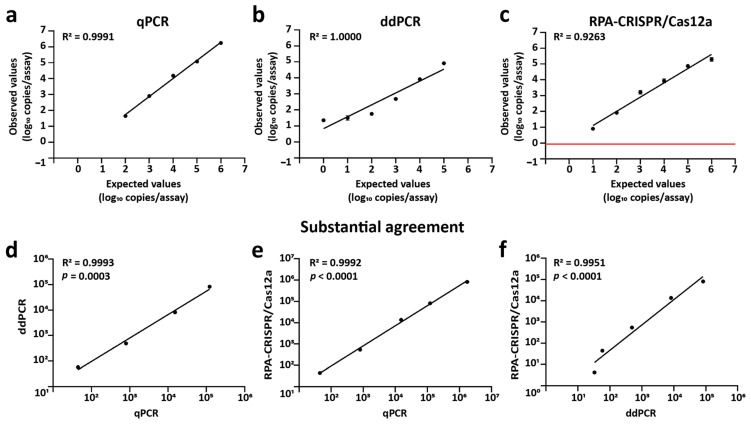
Comparison of qPCR, ddPCR, and RPA-CRISPR/Cas12a methods for cccDNA detection using plasmid standards. Serial dilutions of a plasmid containing cccDNA were analyzed using (**a**) qPCR, (**b**) ddPCR, and (**c**) the RPA-CRISPR/Cas12a system. The red line indicates the limit of detection. Comparative analyses of detection results between (**d**) qPCR and ddPCR, (**e**) qPCR and RPA-CRISPR/Cas12a, and (**f**) ddPCR and RPA-CRISPR/Cas12a show strong correlation, as indicated by R^2^ and *p*-values. Each point and error bar represents the mean and standard deviation of three technical replicates.

cccDNA-containing plasmids were serially diluted and analyzed using qPCR, ddPCR, and RPA-CRISPR/Cas12a. All methods showed strong linear correlation between expected and measured values (qPCR: R^2^ = 0.9991, ddPCR: R^2^ = 1.0000, RPA-CRISPR/Cas12a: R^2^ = 0.9263) within their respective quantification ranges (qPCR: 10^2^–10^6^, ddPCR: 10^0^–10^5^, RPA-CRISPR/Cas12a: 10^1^–10^6^ copies/reaction) (Figure 4a–c). The range of qPCR was selected from the result of gel electrophoresis, which did not display non-specific products (Figure S4), while the range of ddPCR was selected from copy numbers that contain both positive and negative droplets (Figure S5). The limit of detection (LOD) of RPA-CRISPR/Cas12a, defined as the lowest signal reliably distinguishable from background noise, was calculated using the formula 3σ/slope, where σ is the standard deviation of three non-template controls [43] (Figure 4c, red line). All assays had a coefficient of variation (CV%) below 25%, indicating high precision. Correlation between paired methods further validated accuracy: qPCR vs. ddPCR (R^2^ = 0.9993, *p* = 0.0003), qPCR vs. RPA-CRISPR/Cas12a (R^2^ = 0.9992, *p* < 0.0001), and ddPCR vs. RPA-CRISPR/Cas12a (R^2^ = 0.9951, *p* < 0.0001) (Figure 4d–f).

### 2.5. Application to Clinical Liver Samples

**Figure 5:**
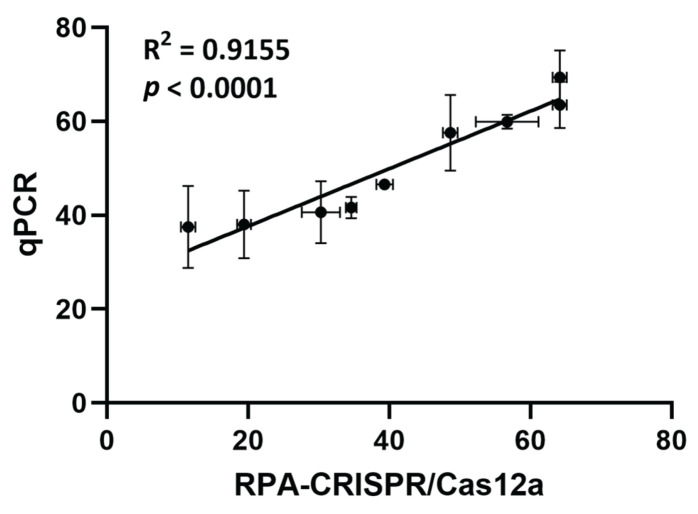
Comparison of RPA-CRISPR/Cas12a to qPCR for quantification of cccDNA. The intrahepatic cccDNA levels were measured by both methods and revealed substantial agreement, indicated by R^2^ and *p*-value. Each point and error bar represents the mean and standard deviation of three technical replicates.

Overt hepatitis B infection is characterized by the presence of HBsAg, indicating active viral replication. In contrast, occult hepatitis B infection (OBI) is defined by the presence of detectable HBV DNA in blood or liver despite the absence of HBsAg [44]. Based on HBsAg status, intrahepatic cccDNA levels were quantified in eight overt HBV and two OBI patients (HBV group) using both qPCR and RPA-CRISPR/Cas12a assays. Liver tissues from six individuals who tested negative for HBsAg, anti-HBc, and anti-HBs served as negative controls (non-HBV group). Baseline clinical and laboratory characteristics of the study participants are summarized in Table 2. No significant differences were observed between HBV and non-HBV groups for most clinical parameters, except for cirrhosis, which was present only in the HBV group. Additionally, no significant differences were found between overt and occult HBV infection subgroups (Table S2). As shown in Table 3, cccDNA was successfully detected in all HBV samples by qPCR (100% sensitivity) and in nine samples by RPA-CRISPR/Cas12a (90% sensitivity), while no cccDNA was detected in non-HBV controls. Notably, the assay enabled cccDNA quantification even in OBI samples with very low cccDNA levels [45]. Comparative analysis revealed a strong linear correlation between RPA-CRISPR/Cas12a and qPCR results (R^2^ = 0.9155, *p* < 0.0001) (Figure 5). These findings demonstrate that the RPA-CRISPR/Cas12a assay provides quantification performance comparable to qPCR and is suitable for accurate measurement of intrahepatic cccDNA.

## 3. Discussion

cccDNA remains the key obstacle to curing HBV infection. Accurate, sensitive, specific, and high-throughput quantification of cccDNA is essential for understanding HBV persistence, evaluating antiviral efficacy, and monitoring therapeutic response [11]. The traditional detection method, Southern blotting (SB), is still regarded as the gold standard because it can distinguish cccDNA from other replicative intermediates. However, SB suffers from poor sensitivity, labor-intensive procedures, and restricted scalability, rendering it impractical for routine clinical or research applications [9,10].

To overcome these limitations, qPCR and ddPCR have been developed. qPCR enables relative quantification with high sensitivity and adaptability to various sample types [46,47]. However, its specificity can be compromised by residual rcDNA, leading to false positives [11,12]. Although enzymatic pretreatment can minimize these signals, it cannot completely eliminate them [9,16]. ddPCR provides absolute quantification with superior sensitivity and precision [17,48,49], but its high cost and technical demands hinder its widespread application [18].

Although qPCR remains a standard diagnostic tool, it also requires a complex instrument, skilled personnel, and costly reagents. These factors restrict its use in resource-limited settings and hinder large-scale screening and post-treatment monitoring [50,51]. In this context, CRISPR-based diagnostics have emerged as promising alternatives due to their programmability, high specificity, and potential for point-of-care implementation [23,26]. CRISPR/Cas-based assays are cost-effective, rapid, and user-friendly alternatives while maintaining diagnostic accuracy and single-nucleotide discrimination comparable to qPCR. Several platforms, such as DETECTR (DNA endonuclease-targeted CRISPR trans reporter) [52], HOLMES (one-HOur Low-cost Multipurpose highly Efficient System) [53], and MiSHERLOCK (the minimally instrumented SHERLOCK) [54] have demonstrated instrument-free detection of viral nucleic acids, enabling sensitive and direct analysis of clinical samples within an hour. The integration of isothermal amplification methods, such as recombinase polymerase amplification (RPA), further enhances assay sensitivity [52,54]. Overall, CRISPR/Cas-based tools represent a next-generation diagnostic approach for sensitive detection and precise quantification of HBV cccDNA, providing a practical alternative to PCR-based techniques.

In 2022, Zhang et al. demonstrated a Cas13a-based method for cccDNA detection [40]. The assay achieved high sensitivity, detecting as few as one copy/µL of HBV cccDNA, and identified cccDNA in 29 of 40 HBV-related liver tissue samples when combined with an amplification step. However, the method required transcription of DNA to RNA, which increased procedural complexity and the risk of RNA degradation. In contrast, our study presents, to our knowledge, the first application of an RPA-CRISPR/Cas12a system for intrahepatic cccDNA quantification. This approach offers several key advantages. It enables the direct detection of double-stranded cccDNA, eliminating the need for RNA intermediates, thereby simplifying the workflow and enhancing assay stability. We designed two crRNAs targeting conserved cccDNA regions across HBV genotypes A–D, which are the predominant genotypes in European countries, North America, Asia, Africa, Australia, and Oceania [55]. This broad compatibility enhances the assay’s utility across global HBV populations. Among these, crRNA1 contained a single mismatch within the seed region (typically the six nucleotides proximal to the PAM), resulting in a weaker fluorescence signal compared to crRNA2v1 and crRNA2v2, both of which had two mismatches outside the seed region. This observation aligns with previous reports indicating that mismatches within the PAM or seed region attenuate the trans-cleavage activity of Cas12a [52].

Our RPA-CRISPR/Cas12a assay demonstrates high specificity, detecting cccDNA exclusively in liver tissue, with no signal observed in plasma or serum. Although HBV cccDNA is typically considered liver-specific [56], some studies have reported its detection in plasma, peripheral blood mononuclear cells (PBMCs), and bone marrow mononuclear cells (MMNCs) [57,58], but these findings remain inconsistent. The assay also exhibits high sensitivity, reliably detecting as few as 10 copies per reaction, and provides a broader dynamic range than qPCR in our study. The quantitative linear range of the RPA-CRISPR/Cas12a assay was comparable to that of ddPCR. Further optimization of reaction conditions may extend its limit of quantification and improve quantitative performance. Moreover, the assay demonstrates a strong correlation with both qPCR and ddPCR results, underscoring its analytical accuracy. Collectively, these findings indicate that the RPA-CRISPR/Cas12a assay is a reliable and effective tool for cccDNA quantification.

Clinically, our RPA-CRISPR/Cas12a assay successfully quantified intrahepatic cccDNA in both overt and occult HBV infection, highlighting its ability to detect extremely low viral reservoirs that often escape conventional serological assays [59]. However, one clinical sample tested negative by RPA-CRISPR/Cas12a but positive by qPCR. This discrepancy is likely attributable to very low cccDNA copy numbers, together with differences in amplification chemistry and target accessibility between the two methods. The RPA-CRISPR/Cas12a mechanism requires intact target recognition by both amplification primers and the crRNA, which may reduce detection sensitivity at extremely low copy numbers.

In overt infection, cccDNA levels were generally higher, consistent with active viral replication and HBsAg positivity. In contrast, detectable but lower cccDNA levels in OBI patients reflect the persistence of transcriptionally silent or weakly active cccDNA within hepatocytes despite HBsAg negativity [44]. This finding supports previous evidence that OBI represents a state of suppressed, rather than cleared, viral replication [44]. The persistence of low-level cccDNA may contribute to HBV reactivation under immunosuppressive conditions [60] and promote hepatocarcinogenesis through low-grade viral transcription [61]. The ability of the RPA-CRISPR/Cas12a assay to detect cccDNA even at very low copy numbers, as observed in OBI samples, highlights its potential utility in HBV research, particularly for accurate cccDNA quantification in mechanistic studies, therapeutic evaluation, and biomarker validation.

Despite its promising performance, certain limitations should be noted. First, although cccDNA was successfully quantified in both HBsAg-positive and HBsAg-negative samples, the number of clinical specimens in this study was limited due to the restricted availability of patients undergoing surgical tumor resection. Further evaluation in a larger cohort, particularly in HBsAg-negative individuals, is necessary to validate assay performance. Second, we did not directly confirm cccDNA extraction using SB due to its labor-intensive nature. Although our extraction and enzymatic pretreatment protocol has been previously validated by SB [17], direct confirmation in our study would strengthen the specificity of cccDNA detection and rule out potential rcDNA cross-reactivity. Nevertheless, qPCR was employed as a practical comparator for assay validation.

For future clinical translation, the RPA-CRISPR/Cas12a assay will require analytical validation and standardization to ensure robustness and inter-laboratory comparability, followed by multicenter studies to confirm diagnostic performance and clinical thresholds. Integration into automated or microfluidic platforms, which has previously been demonstrated for the detection of *Staphylococcus aureus* [62] and SARS-CoV-2 virus [63,64,65], would further enhance scalability, reproducibility, and suitability for high-throughput clinical use. Collectively, these steps will facilitate the transition of this platform from a research assay to a clinically applicable diagnostic tool for confirming complete HBV cure in the future.

## 4. Materials and Methods

### 4.1. Patient Samples

A total of 10 patients with HBV-associated hepatocellular carcinoma (HCC) and 6 non-HBV patients who underwent surgical tumor resection were enrolled in this study. Among the non-HBV group, one patient had alcoholic liver disease with hepatitis C virus (HCV)-associated HCC, while the remaining five had colorectal liver metastases (CRLM). Non-tumor liver tissues were collected at King Chulalongkorn Memorial Hospital, Thailand, and preserved in RNAlater™ Stabilization Solution (Invitrogen, Carlsbad, CA, USA). All samples were stored at −80 °C until analysis.

### 4.2. Plasmids

Plasmids containing monomeric HBV genomes (pHBV) from genotypes A (NCBI: AB246337), B (NCBI: AB246342), C (NCBI: AB246344), and D (NCBI: AB246347) were kindly provided by Dr. Mizokami, Department of Clinical Molecular Informative Medicine, Nagoya City University Graduate School of Medical Sciences, Japan [66]. As HBV genotype C is the predominant strain in Southeast Asia [55], the plasmid containing genotype C was used in relevant experiments.

### 4.3. HBsAg, Anti-HBc, and Anti-HBs Testing

Serum HBsAg, anti-HBc, and anti-HBs levels were measured using commercial diagnostic assays. HBsAg was quantified using a standard assay (Abbott Laboratories, Chicago, IL, USA; cut-off: 0.05 IU/mL). Anti-HBc and anti-HBs levels were determined via electrochemiluminescence immunoassay (ECLIA; Roche Diagnostics, Berkeley, CA, USA), with a negative cut-off defined as >1.0 COI (cutoff index) for anti-HBc and <10 IU/L for anti-HBs.

### 4.4. Genomic DNA Extraction from Serum and Plasma

Genomic DNA was extracted from patient serum and plasma samples using the GenUP™ gDNA Kit (Biotechrabbit, Berlin, Germany), following the manufacturer’s instructions. Briefly, 50 μL of the sample was mixed with 200 μL LYSIS LG buffer, 25 μL Proteinase K (25 mg/mL), and 3 μL RNase A (100 mg/mL), incubated at 50 °C for 30 min, then processed through a silica-based spin column system. DNA was eluted in 50 μL of elution buffer and stored at −20 °C until use.

### 4.5. Protein-Free DNA Extraction from Liver Tissue

Protein-free DNA (pf-DNA) was extracted using a modified Hirt extraction procedure [17]. Briefly, 25 mg of liver tissue was lysed in 2.5 mL of lysis buffer (10 mM Tris-HCl, pH 8.0; 10 mM EDTA; 10 mM NaCl; 0.5% SDS) and incubated overnight at 37 °C with gentle rotation. Lysates were extracted with an equal volume of Tris-saturated phenol, vortexed, and rotated for 30 min at room temperature. After centrifugation (2500 rpm, 10 min, 4 °C), the upper aqueous phase was collected and re-extracted with Tris-saturated phenol and chloroform:isoamyl alcohol (49:1) and centrifugation for 30 min. DNA was precipitated with 0.1 volume of 3 M sodium acetate and 2.5 volumes of cold absolute ethanol, followed by incubation at −80 °C for 1 h and centrifugation (4000 rpm, 15 min, 4 °C). The DNA pellet was washed with 70% ethanol, centrifuged again, air-dried, and resuspended in 100 μL of TE buffer (10 mM Tris-HCl, pH 8.0; 1 mM EDTA) overnight at 4 °C. pf-DNA was treated with 6 μL of RNase A (Invitrogen, Carlsbad, CA, USA) and stored at −20 °C.

### 4.6. cccDNA Enrichment by Enzymatic Digestion

To enrich for cccDNA, genomic DNA or pf-DNA was first digested with *Hind*III, which does not cleave within cccDNA sequences [40], to eliminate host genomic DNA. The digested DNA was subsequently treated with 10 U of plasmid-safe ATP-dependent DNase (PSAD; Epicentre, New York City, NY, USA) to remove rcDNA, ssDNA, and dsDNA. Digestion reactions were incubated at 37 °C for 30 min.

### 4.7. Quantification of HBV cccDNA by qPCR

cccDNA was quantified by qPCR using primers that span the single-stranded gap region of rcDNA, enabling selective amplification of cccDNA [17]. The forward sequence was 5′-ACGGGGCGCACCTCTCTTTACGCGG-3′ (nt 1519–1543), the reverse sequence was 5′-CAAGGCACAGCTTGGAGGCTTGAAC-3′ (nt 1862–1886), and the probe sequence was 5′-FAM-AACGACCGACCTTGAGGCAT-MGB-3′. Reactions were performed in a 10 μL total volume included 1000 ng of pre-digested DNA (measured before enzymatic digestion), 2.5 μL CAPITAL qPCR Probe Master Mix (Biotechrabbit, Berlin, Germany), 0.3 μM primers, and 0.1 μM probe. Amplification was performed using a StepOnePlus™ Real-Time PCR System (Thermo Fisher Scientific, Waltham, MA, USA) with the following conditions: 95 °C for 3 min, followed by 50 cycles of 95 °C for 15 s and 65 °C for 30 s. Quantification was achieved using a standard curve derived from serial dilutions of pHBV plasmid (AB246344). Values below detection thresholds were labeled as undetermined.

### 4.8. Quantification of HBV cccDNA by ddPCR

Droplet digital PCR (ddPCR) was performed, following the previous report [17], using the QX200 system (Bio-Rad, Hercules, CA, USA). Each reaction contained 2× ddPCR Supermix for Probes (no dUTP), 900 nM primers, 250 nM probe, 1 U of *Hae*III, plasmid DNA template. Droplets were generated using the QX200 Droplet Generator. Thermal cycling was carried out using the C1000 Touch Thermal Cycler under the following conditions: 95 °C for 10 min; 40 cycles of 94 °C for 30 s and 61.2 °C for 1 min; and 98 °C for 10 min. Droplets were read on the QX200 Droplet Reader and analyzed using QuantaSoft v1.7 (Bio-Rad, Hercules, CA, USA).

### 4.9. Recombinase Polymerase Amplification (RPA)

RPA reactions were performed using the TwistAmp Basic Kit (TwistDx, Berkshire, UK). Each 50 μL reaction contained 100 nM of each primer, 14 mM MgOAc, 29.5 μL rehydration buffer, lyophilized enzyme pellet, and 1000 ng of pre-digested DNA (measured before enzymatic digestion). Reactions were incubated at 39 °C for 50 min, followed by heat inactivation at 75 °C for 5 min.

### 4.10. CRISPR/Cas12a Fluorescence Assay

CRISPR/Cas12a-based detection was conducted using EnGen^®^ Lba Cas12a (NEB, Ipswich, MA, USA), synthetic crRNAs and ssDNA fluorescent reporters (BIONICS, Seoul, Republic of Korea). Final 100 μL reactions contained: 1× NEBuffer r2.1, 30 nM Cas12a, 30 nM crRNA, 300 nM reporter, 50 μL of RPA product or HBV plasmid template. Cas12a ribonucleoprotein complexes were preassembled by incubating Cas12a with crRNA for 15 min at room temperature. DNA template and reporter were then added, and reactions were incubated at 37 °C in black 96-well plates. For kinetics experiments, fluorescence was recorded every 5 min using a Synergy HTX Multimode Reader (BioTek, Shoreline, WA, USA) at excitation/emission wavelengths of 483/530 nm. Quantification was achieved by converting RFU values into copy numbers using a standard curve derived from serial dilutions of the pHBV plasmid (AB246344). The non-template control (NTC) was used to determine the detection threshold. Samples with RFU below the NTC were labeled as undetermined.

### 4.11. Statistical Analysis

Statistical analyses were performed using GraphPad Prism version 10.3.1 (GraphPad, Inc., Boston, MA, USA). Data are presented as means and standard deviations (SD). Mean differences were determined using two-way ANOVA followed by Tukey’s multiple comparisons test. Linear regression was performed on log-transformed data to calculate the coefficient of determination (R^2^). Correlations between two continuous variables were analyzed using Pearson’s correlation coefficient with a 95% confidence interval. Baseline characteristics between groups were compared using Fisher’s exact test for categorical variables (presented as number and percentage) and unpaired t-test for continuous variables.

## 5. Conclusions

In summary, the RPA-CRISPR/Cas12a assay represents a novel, accurate, and scalable platform for cccDNA quantification. With further refinement, it holds the potential to complement or even replace qPCR and ddPCR in both research and clinical settings, particularly for clinical and therapeutic monitoring in HBV research in resource-constrained environments.

## Figures and Tables

**Table 1:** Sequence of crRNAs for CRISPR/Cas12a assay.

crRNA	Position/Strand	*PAM* ^a^ Guide Sequence (5′–3′)	Predicted Efficiency	Off-Targets for 0-1-2-3-4 Mismatched + next to PAM ^c^	Location	Matched Genotypes
crRNA1	1539/fw	*TTTA* CGCGG**T**CTCCCCGTCTGTGCCTT ^b^	52	0-0-0-0-0 0-0-0-0-0 0 off-targets	100 nt upstream of DR2	A, C
crRNA2v1 (crRNA2)	1764/rev	*TTTA* TGCCTACAGCCTCC**T**A**G**TACAAA ^b^	79	0-0-0-0-0 0-0-0-0-0 0 off-targets	50 nt upstream of the terminal	B, D
crRNA2v2		*TTTA* TGCCTACAGCCTCC**C**A**G**TACAAA ^b^				C
Negative	-	*Not applicable* CGTTAATCGCGTATAATACGG	-	-	-	-

The predicted efficiency and off-targets were analyzed by CRISPOR. Matched genotypes were analyzed by Jalview 2.11.5.1. ^a^ Italic and ^b^ bold type characters indicate PAM sequences and mismatches presented in crRNAs, respectively. ^c^ Grey characters indicate the number of off-targets at the position next to PAM sequence.

**Table 2:** Baseline characteristics of patients with HBV and Non-HBV.

Characteristics	HBV (n = 10)	Non-HBV (n = 6)	*p*
Age (Years)	61.7 ± 8.5	62.3 ± 15.9	0.9181
Female (%)	3 (30)	3 (50)	0.6066
Hemoglobin (g/dL)	11.2 ± 1.7	11.4 ± 1.8	0.7973
Platelet count (10^3^/µL)	155.4 ± 120.5	180.8 ± 45.2	0.631
Total bilirubin (mg/dL)	1.2 ± 0.7	1.0 ± 0.4	0.5878
AST (IU/L)	177.3 ± 174.3	428.2 ± 410.6	0.1074
ALT (IU/L)	163.8 ± 148.8	260.3 ± 271.8	0.3693
ALP (IU/L)	83.8 ± 45.3	97.7 ± 30.0	0.5181
Albumin (mg/dL)	3.3 ± 0.5	3.4 ± 0.7	0.5852
INR	1.3 ± 0.2	1.1 ± 0.1	0.1914
AFP (ng/mL)	15.5 ± 28.8	6.0 ± 5.6	0.6713
Cirrhosis (Yes)	7 (77.78)	0 (0)	0.0070 *

Data expressed as mean ± standard deviation (SD) or n (%), * *p* < 0.05, AST: aspartate aminotransaminase, ALT: alanine aminotransaminase, ALP: alkaline phosphatase, AFP: alpha-fetoprotein, INR: International normalized ratio.

**Table 3:** Results of cccDNA quantification by qPCR and RPA-CRISPR/Cas12a in clinical samples.

Samples	Overt/Occult	HBV Viral Load (IU/mL)	HBsAg	HBsAg Quantitative (IU/mL)	Anti-HBc	Anti-HBs	qPCR (Copies/1000 ng gDNA)	RPA-CRISPR/Cas12a (Copies/1000 ng gDNA)
HBV 1	Overt	337,391	+	58	+	+	69.35	64.15
HBV 2	Overt	34,419	+	1691.10	+	−	63.47	56.68
HBV 3	Overt	1325	+	32.93	+	−	57.57	45.47
HBV 4	Overt	<10	+	62.57	+	−	38.11	14.99
HBV 5	Occult	398	−	<0.02	+	−	46.54	34.61
HBV 6	Overt	376	+	35.97	+	−	41.66	30.3
HBV 7	Overt	216	+	1130.97	+	−	40.61	24.65
HBV 8	Overt	1340	+	2316.45	+	−	59.91	48.62
HBV 9	Occult	<10	−	0.04	+	+	37.49	11.47
HBV 10	Overt	<10	+	0.13	+	+	28.43	UD
Non-HBV 1	ND	ND	−	<0.02	−	−	UD	UD
Non-HBV 2	ND	ND	−	<0.02	−	−	UD	UD
Non-HBV 3	ND	ND	−	<0.02	−	−	UD	UD
Non-HBV 4	ND	ND	−	<0.02	−	−	UD	UD
Non-HBV 5	ND	ND	−	<0.02	−	−	UD	UD
Non-HBV 6	ND	ND	−	<0.02	−	−	UD	UD

+, −, ND, and UD mean positive, negative, not determined, and undetermined, respectively.

## Data Availability

The original contributions presented in this study are included in the article/Supplementary Material.

## Supplementary Materials

The following supporting information can be downloaded at: https://www.mdpi.com/article/10.3390/ijms27010551/s1.

## Abbreviations

AFP	Alpha-fetoprotein
ALP	Alkaline phosphatase
ALT	Alanine aminotransferase
AST	Aspartate aminotransferase
Cas12a	CRISPR-associated protein 12a
cccDNA	Covalently closed circular DNA
CHB	Chronic HBV infection
CRISPR	Clustered regularly interspaced short palindromic repeats
CRLM	Colorectal liver metastasis
crRNA	CRISPR RNA
CV	Coefficient of variation
ddPCR	Droplet digital PCR
DR	Direct repeat
dsDNA	Double-stranded DNA
HBsAg	Hepatitis B surface antigen
HBV	Hepatitis B virus
HCC	Hepatocellular carcinoma
HCV	Hepatitis C virus
IFNs	Interferons
INR	International normalized ratio
LOD	Limit of detection
MMNCs	Bone marrow mononuclear cells
NAs	Nucleos(t)ide analogues
NTC	Non-template control
OBI	Occult hepatitis B infection
PAM	Protospacer adjacent motif
PBMCs	Peripheral blood mononuclear cells
pf-rcDNA	Protein-free rcDNA
pgRNA	Pregenomic RNA
POCT	Point-of-care testing
PSAD	Plasmid-Safe ATP-Dependent DNase
qPCR	Quantitative PCR
rcDNA	Relaxed circular DNA
RIs	Replicative intermediates
RPA	Recombinase polymerase amplification
SB	Southern blotting
SD	Standard deviations
ssDNA	Single-stranded DNA
WHO	World Health Organization

## References

[1] World Health Organization (2024). Global Hepatitis Report 2024: Action for Access in Low-and Middle-Income Countries.

[2] Hsu Y.-C., Huang D.Q., Nguyen M.H. (2023). Global burden of hepatitis B virus: Current status, missed opportunities and a call for action. Nat. Rev. Gastroenterol. Hepatol..

[3] Dandri M., Petersen J. (2020). cccDNA Maintenance in Chronic Hepatitis B—Targeting the Matrix of Viral Replication. Infect. Drug Resist..

[4] Nguyen M.H., Wong G., Gane E., Kao J.-H., Dusheiko G. (2020). Hepatitis B Virus: Advances in Prevention, Diagnosis, and Therapy. Clin. Microbiol. Rev..

[5] From Fungus to Virus, a Phase 1b Clinical Trial Investigating the Safety and Efficacy of Terbinafine in Chronic Hepatitis B Patients. https://clinicaltrials.gov/study/NCT06295328.

[6] A Phase 1, Open-Label, First-in-Human, Dose Escalation (Part 1) and Expansion (Part 2) Study to Evaluate the Safety, Tolerability, Pharmacokinetics, and Antiviral Activity of PBGENE-HBV in Participants with Chronic Hepatitis B (ELIMINATE-B). https://clinicaltrials.gov/study/NCT06680232.

[7] Saitta C., Pollicino T., Raimondo G. (2022). Occult Hepatitis B Virus Infection: An Update. Viruses.

[8] Li X., Zhao J., Yuan Q., Xia N. (2017). Detection of HBV Covalently Closed Circular DNA. Viruses.

[9] Allweiss L., Testoni B., Yu M., Lucifora J., Ko C., Qu B., Lütgehetmann M., Guo H., Urban S., Fletcher S.P. (2023). Quantification of the hepatitis B virus cccDNA: Evidence-based guidelines for monitoring the key obstacle of HBV cure. Gut.

[10] Tu T., Zehnder B., Qu B., Ni Y., Main N., Allweiss L., Dandri M., Shackel N., George J., Urban S. (2020). A novel method to precisely quantify hepatitis B virus covalently closed circular (ccc)DNA formation and maintenance. Antivir. Res..

[11] Zhang H., Tu T. (2021). Approaches to quantifying hepatitis B virus covalently closed circular DNA. Clin. Mol. Hepatol..

[12] Xia Y., Stadler D., Ko C., Protzer U. (2016). Analyses of HBV cccDNA quantification and modification. Hepatitis B Virus: Methods and Protocols.

[13] Luo J., Cui X., Gao L., Hu J. (2017). Identification of an intermediate in hepatitis B virus covalently closed circular (CCC) DNA formation and sensitive and selective CCC DNA detection. J. Virol..

[14] Werle–Lapostolle B., Bowden S., Locarnini S., Wursthorn K., Petersen J., Lau G., Trepo C., Marcellin P., Goodman Z., Delaney W.E. (2004). Persistence of cccDNA during the natural history of chronic hepatitis B and decline during adefovir dipivoxil therapy. Gastroenterology.

[15] Wang Z., Chen Y., Deng H., Zhen X., Xiong J., Hu Y. (2022). Quantification of intrahepatic cccDNA in HBV associated hepatocellular carcinoma by improved ddPCR method. J. Virol. Methods.

[16] Qu B., Ni Y., Lempp F.A., Vondran F.W.R., Urban S. (2018). T5 Exonuclease Hydrolysis of Hepatitis B Virus Replicative Intermediates Allows Reliable Quantification and Fast Drug Efficacy Testing of Covalently Closed Circular DNA by PCR. J. Virol..

[17] Hayashi S., Isogawa M., Kawashima K., Ito K., Chuaypen N., Morine Y., Shimada M., Higashi-Kuwata N., Watanabe T., Tangkijvanich P. (2022). Droplet digital PCR assay provides intrahepatic HBV cccDNA quantification tool for clinical application. Sci. Rep..

[18] Kojabad A.A., Farzanehpour M., Galeh H.E.G., Dorostkar R., Jafarpour A., Bolandian M., Nodooshan M.M. (2021). Droplet digital PCR of viral DNA/RNA, current progress, challenges, and future perspectives. J. Med. Virol..

[19] Sorek R., Kunin V., Hugenholtz P. (2008). CRISPR—A widespread system that provides acquired resistance against phages in bacteria and archaea. Nat. Rev. Microbiol..

[20] Horvath P., Barrangou R. (2010). CRISPR/Cas, the immune system of bacteria and archaea. Science.

[21] Datsenko K.A., Pougach K., Tikhonov A., Wanner B.L., Severinov K., Semenova E. (2012). Molecular memory of prior infections activates the CRISPR/Cas adaptive bacterial immunity system. Nat. Commun..

[22] Yuan B., Yuan C., Li L., Long M., Chen Z. (2022). Application of the CRISPR/Cas system in pathogen detection: A review. Molecules.

[23] Li P., Wang L., Yang J., Di L.-J., Li J. (2021). Applications of the CRISPR-Cas system for infectious disease diagnostics. Expert Rev. Mol. Diagn..

[24] Zhou B., Yang R., Sohail M., Kong X., Zhang X., Fu N., Li B. (2023). CRISPR/Cas14 provides a promising platform in facile and versatile aptasensing with improved sensitivity. Talanta.

[25] Agha A.S.A., Al-Samydai A., Aburjai T. (2025). New frontiers in CRISPR: Addressing antimicrobial resistance with Cas9, Cas12, Cas13, and Cas14. Heliyon.

[26] Aman R., Mahas A., Mahfouz M. (2020). Nucleic acid detection using CRISPR/Cas biosensing technologies. ACS Synth. Biol..

[27] Wang M., Wang H., Li K., Li X., Wang X., Wang Z. (2023). Review of CRISPR/Cas systems on detection of nucleotide sequences. Foods.

[28] Varble A., Marraffini L.A. (2019). Three new Cs for CRISPR: Collateral, communicate, cooperate. Trends Genet..

[29] Zhou J., Li Z., Olajide J.S., Wang G. (2024). CRISPR/Cas-based nucleic acid detection strategies: Trends and challenges. Heliyon.

[30] Fozouni P., Son S., de León Derby M.D., Knott G.J., Gray C.N., D’Ambrosio M.V., Zhao C., Switz N.A., Kumar G.R., Stephens S.I. (2021). Amplification-free detection of SARS-CoV-2 with CRISPR-Cas13a and mobile phone microscopy. Cell.

[31] Ali Z., Sánchez E., Tehseen M., Mahas A., Marsic T., Aman R., Sivakrishna Rao G., Alhamlan F.S., Alsanea M.S., Al-Qahtani A.A. (2021). Bio-SCAN: A CRISPR/dCas9-based lateral flow assay for rapid, specific, and sensitive detection of SARS-CoV-2. ACS Synth. Biol..

[32] Wang B., Wang R., Wang D., Wu J., Li J., Wang J., Liu H., Wang Y. (2019). Cas12aVDet: A CRISPR/Cas12a-Based Platform for Rapid and Visual Nucleic Acid Detection. Anal. Chem..

[33] Wang H., Wu Q., Zhou M., Li C., Yan C., Huang L., Qin P. (2022). Development of a CRISPR/Cas9-integrated lateral flow strip for rapid and accurate detection of Salmonella. Food Control.

[34] Samanta D., Ebrahimi S.B., Ramani N., Mirkin C.A. (2022). Enhancing CRISPR-Cas-Mediated Detection of Nucleic Acid and Non-nucleic Acid Targets Using Enzyme-Labeled Reporters. J. Am. Chem. Soc..

[35] Liu H., Chang S., Chen S., Du Y., Wang H., Wang C., Xiang Y., Wang Q., Li Z., Wang S. (2022). Highly sensitive and rapid detection of SARS-CoV-2 via a portable CRISPR-Cas13a-based lateral flow assay. J. Med. Virol..

[36] Li P.-R., Wang Z.-X., Xu Z.-K., Wang J., Li B., Shen X., Xu Z.-L. (2024). An RPA-assisted CRISPR/Cas12a assay combining fluorescence and lateral flow strips for the rapid detection of enterotoxigenic Bacillus cereus. J. Agric. Food Chem..

[37] Yin L., Duan N., Chen S., Yao Y., Liu J., Ma L. (2021). Ultrasensitive pathogenic bacteria detection by a smartphone-read G-quadruplex-based CRISPR-Cas12a bioassay. Sens. Actuators B Chem..

[38] Du Y., Ji S., Dong Q., Wang J., Han D., Gao Z. (2023). Amplification-free detection of HBV DNA mediated by CRISPR-Cas12a using surface-enhanced Raman spectroscopy. Anal. Chim. Acta.

[39] Zhao C., Du L., Hu J., Hou X. (2024). Recombinase polymerase amplification and target-triggered CRISPR/Cas12a assay for sensitive and selective Hepatitis B virus DNA analysis based on lanthanide tagging and inductively coupled plasma mass spectrometric detection. Anal. Chem..

[40] Zhang X., Tian Y., Xu L., Fan Z., Cao Y., Ma Y., Li H., Ren F. (2022). CRISPR/Cas13-assisted hepatitis B virus covalently closed circular DNA detection. Hepatol. Int..

[41] Concordet J.-P., Haeussler M. (2018). CRISPOR: Intuitive guide selection for CRISPR/Cas9 genome editing experiments and screens. Nucleic Acids Res..

[42] Waterhouse A.M., Procter J.B., Martin D.M.A., Clamp M., Barton G.J. (2009). Jalview Version 2—A multiple sequence alignment editor and analysis workbench. Bioinformatics.

[43] Qiao J., Zhang J., Jiang Q., Jin S., He R., Qiao B., Liu Y. (2025). Boosting CRISPR/Cas12a intrinsic RNA detection capability through pseudo hybrid DNA–RNA substrate design. Nucleic Acids Res..

[44] Raimondo G., Locarnini S., Pollicino T., Levrero M., Zoulim F., Lok A.S., Allain J.-P., Berg T., Bertoletti A., Brunetto M.R. (2019). Update of the statements on biology and clinical impact of occult hepatitis B virus infection. J. Hepatol..

[45] Tantiwetrueangdet A., Panvichian R., Sornmayura P., Sueangoen N., Leelaudomlipi S. (2018). Reduced HBV cccDNA and HBsAg in HBV-associated hepatocellular carcinoma tissues. Med. Oncol..

[46] Guo Y., Sheng S., Nie B., Tu Z. (2015). Development of magnetic capture hybridization and quantitative polymerase chain reaction for hepatitis B virus covalently closed circular DNA. Hepat. Mon..

[47] He M.-L., Wu J., Chen Y., Lin M.C., Lau G.K., Kung H.-f. (2002). A new and sensitive method for the quantification of HBV cccDNA by real-time PCR. Biochem. Biophys. Res. Commun..

[48] Huang J.-T., Yang Y., Hu Y.-M., Liu X.-H., Liao M.-Y., Morgan R., Yuan E.-F., Li X., Liu S.-M. (2018). A Highly Sensitive and Robust Method for Hepatitis B Virus Covalently Closed Circular DNA Detection in Single Cells and Serum. J. Mol. Diagn..

[49] Mu D., Yan L., Tang H., Liao Y. (2015). A sensitive and accurate quantification method for the detection of hepatitis B virus covalently closed circular DNA by the application of a droplet digital polymerase chain reaction amplification system. Biotechnol. Lett..

[50] Kumar A., Combe E., Mougené L., Zoulim F., Testoni B. (2024). Applications of CRISPR/Cas as a toolbox for hepatitis B virus detection and therapeutics. Viruses.

[51] Pan Z., Xu L., Fan Z., Ren F. (2025). CRISPR-Cas for hepatitis virus: A systematic review and meta-analysis of diagnostic test accuracy studies. Front. Microbiol..

[52] Chen J.S., Ma E., Harrington L.B., Da Costa M., Tian X., Palefsky J.M., Doudna J.A. (2018). CRISPR-Cas12a target binding unleashes indiscriminate single-stranded DNase activity. Science.

[53] Li S.-Y., Cheng Q.-X., Wang J.-M., Li X.-Y., Zhang Z.-L., Gao S., Cao R.-B., Zhao G.-P., Wang J. (2018). CRISPR-Cas12a-assisted nucleic acid detection. Cell Discov..

[54] De Puig H., Lee R.A., Najjar D., Tan X., Soenksen L.R., Angenent-Mari N.M., Donghia N.M., Weckman N.E., Ory A., Ng C.F. (2021). Minimally instrumented SHERLOCK (miSHERLOCK) for CRISPR-based point-of-care diagnosis of SARS-CoV-2 and emerging variants. Sci. Adv..

[55] Velkov S., Ott J.J., Protzer U., Michler T. (2018). The global hepatitis B virus genotype distribution approximated from available genotyping data. Genes.

[56] Singh M., Dicaire A., Wakil A.E., Luscombe C., Sacks S.L. (2004). Quantitation of hepatitis B virus (HBV) covalently closed circular DNA (cccDNA) in the liver of HBV-infected patients by LightCycler™ real-time PCR. J. Virol. Methods.

[57] Xu C.-H., Li Z.-S., Dai J.-Y., Zhu H.-Y., Yu J.-W. (2011). Nested real-time quantitative polymerase chain reaction assay for detection of hepatitis B virus covalently closed circular DNA. Chin. Med. J..

[58] Takkenberg R., Menting S., Beld M. (2012). Validation of a sensitive and specific real-time PCR for detection and quantitation of hepatitis B virus covalently closed circular DNA in plasma of chronic hepatitis B patients. Diagnosis of Sexually Transmitted Diseases: Methods and Protocols.

[59] He P., Zhang P., Fang Y., Han N., Yang W., Xia Z., Zhu Y., Zhang Z., Shen J. (2023). The role of HBV cccDNA in occult hepatitis B virus infection. Mol. Cell. Biochem..

[60] Pawłowska M., Flisiak R., Gil L., Horban A., Hus I., Jaroszewicz J., Lech-Marańda E., Styczyński J. (2019). Prophylaxis of hepatitis B virus (HBV) infection reactivation–recommendations of the Working Group for prevention of HBV reactivation. Clin. Exp. Hepatol..

[61] Bianca C., Sidhartha E., Tiribelli C., El-Khobar K.E., Sukowati C.H. (2022). Role of hepatitis B virus in development of hepatocellular carcinoma: Focus on covalently closed circular DNA. World J. Hepatol..

[62] Lu L., Zhang H., Lin F., Zhou L., Zhu Z., Yang C. (2023). Sensitive and automated detection of bacteria by CRISPR/Cas12a-assisted amplification with digital microfluidics. Sens. Actuators B Chem..

[63] Chen Y., Zong N., Ye F., Mei Y., Qu J., Jiang X. (2022). Dual-CRISPR/Cas12a-assisted RT-RAA for ultrasensitive SARS-CoV-2 detection on automated centrifugal microfluidics. Anal. Chem..

[64] Xu J., Ma Y., Song Z., Sun W., Liu Y., Shu C., Hua H., Yang M., Liang Q. (2023). Evaluation of an automated CRISPR-based diagnostic tool for rapid detection of COVID-19. Heliyon.

[65] Chen F.-E., Lee P.-W., Trick A.Y., Park J.S., Chen L., Shah K., Mostafa H., Carroll K.C., Hsieh K., Wang T.-H. (2021). Point-of-care CRISPR-Cas-assisted SARS-CoV-2 detection in an automated and portable droplet magnetofluidic device. Biosens. Bioelectron..

[66] Sugiyama M., Tanaka Y., Kato T., Orito E., Ito K., Acharya S.K., Gish R.G., Kramvis A., Shimada T., Izumi N. (2006). Influence of hepatitis B virus genotypes on the intra-and extracellular expression of viral DNA and antigens. Hepatology.

