# Supplementary material for: Development of a Sensitive and Specific RPA-CRISPR/Cas12a Assay for Intrahepatic Quantification of HBV cccDNA
Source: Int J Mol Sci. 2026 Jan 5;27(1):551. doi: 10.3390/ijms27010551 (PMC12786411; doi:10.3390/ijms27010551)
Supplement: Supplementary file 1 [file ijms-27-00551-s001.zip › ijms-4062616-supplementary.pdf]

Supplementary Materials

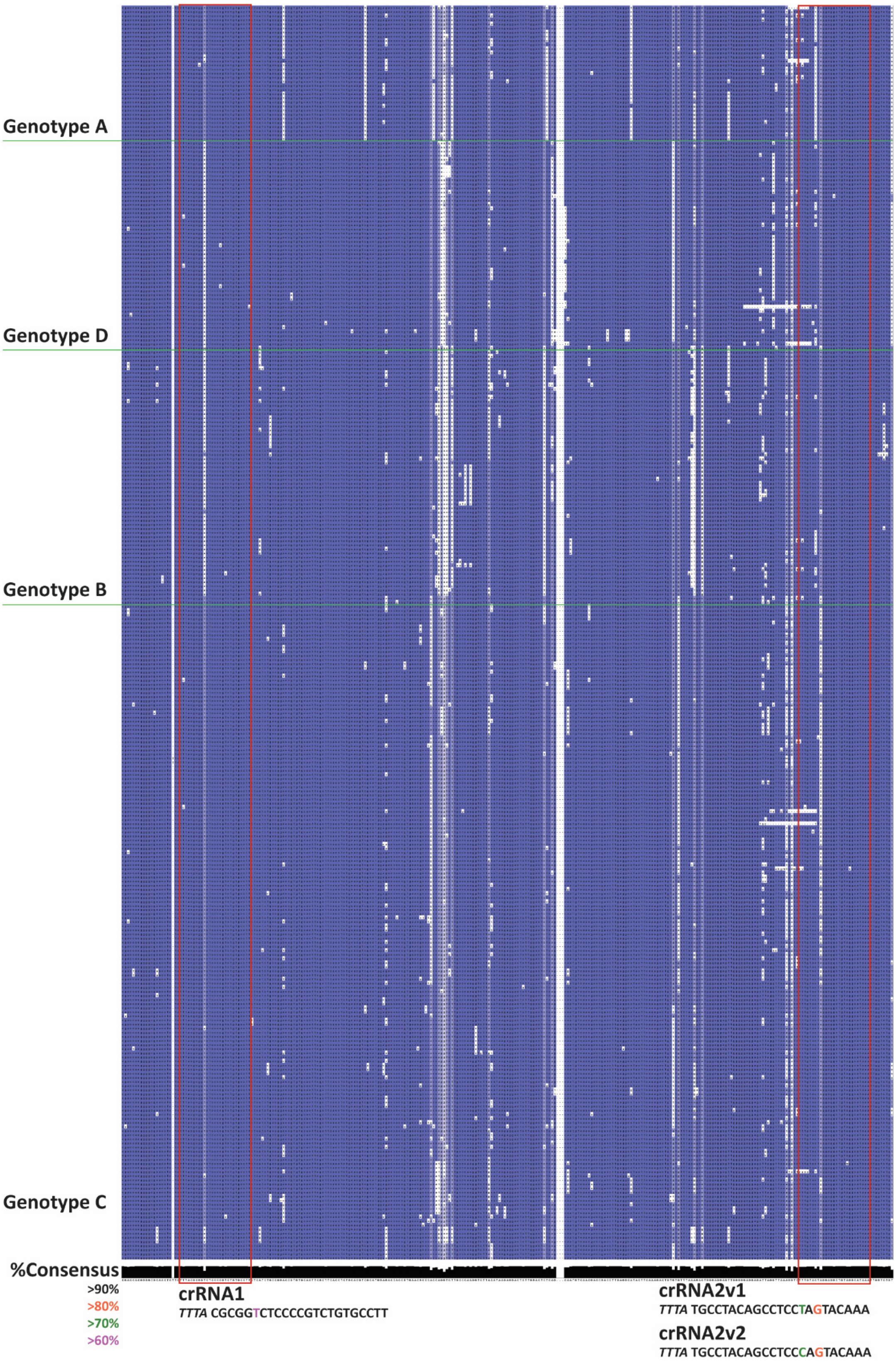

Figure S1. Sequence alignment of the target region in HBV DNA. Complete HBV genome sequences from genotypes A-D were downloaded from NCBI and aligned using Jalview. The sequences corresponding to the designed crRNAs are shown below the alignment. Nucleotide colors indicate the level of sequence consensus among the genotypes.

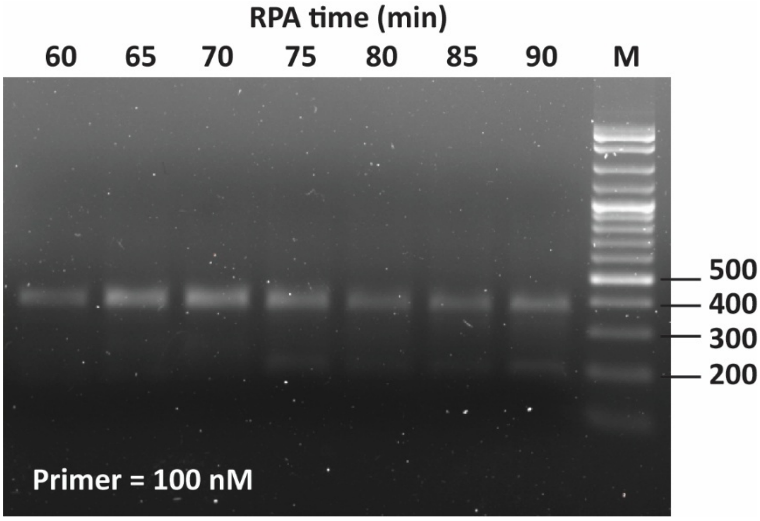

Figure S2. Effect of RPA incubation time on amplification efficiency. Prolonged RPA incubation times from 65 to 90 minutes were tested. Primer concentrations refer to each forward or reverse primer. The expected cccDNA-specific amplicon is 344 bp. M = 100 bp DNA ladder.

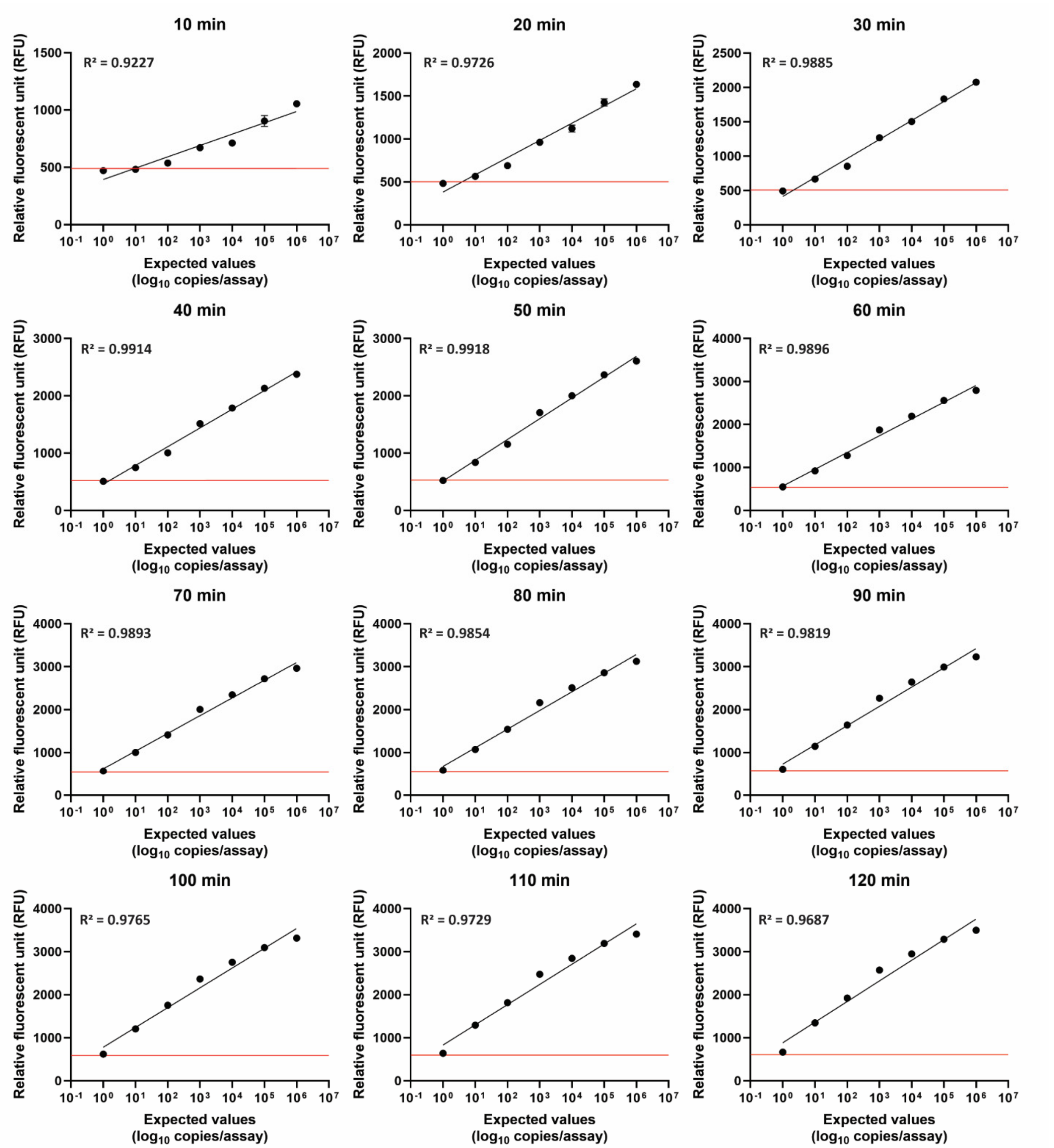

Figure S3. Linear regression analysis of fluorescence intensity versus template copy number. Fluorescence intensities were plotted against template copy numbers at various time points to assess the detection performance. Red lines indicate the limit of detection. R^2 represents the coefficient of determination.

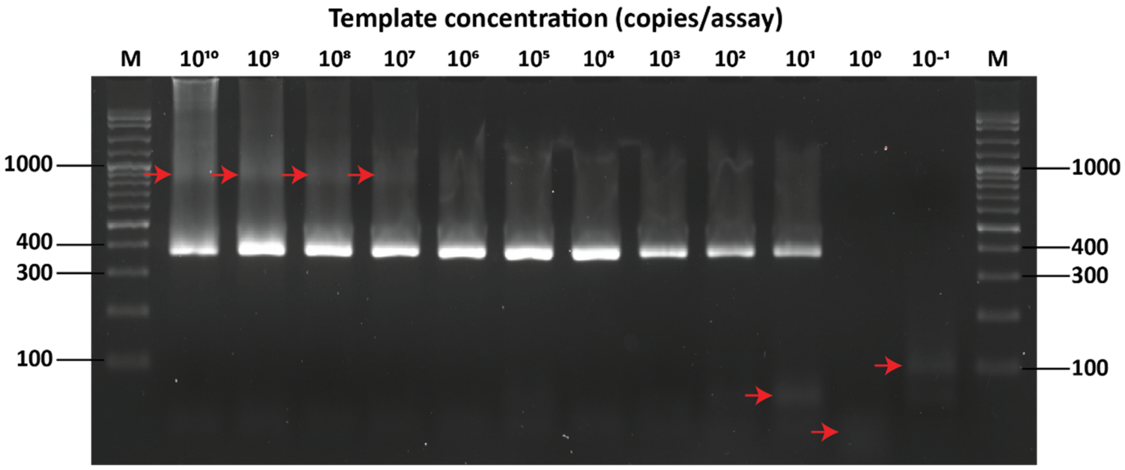

Figure S4. The qPCR products undergo agarose gel electrophoresis at varying template copies. The expected cccDNA-specific amplicon is 344 bp. Red arrows indicate non-specific products. M = 100 bp marker.

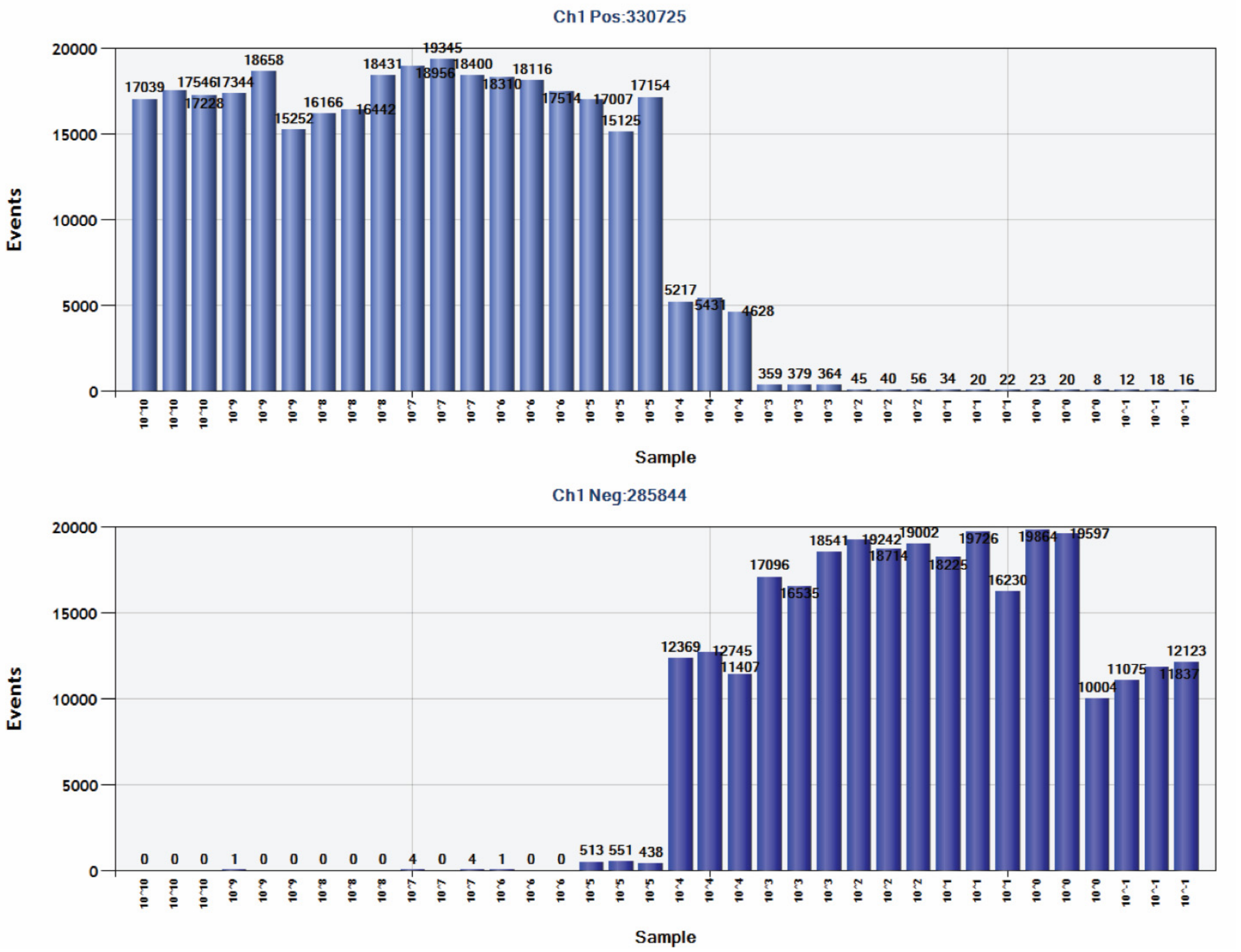

Figure S5. The positive events (upper) and negative events (lower) of ddPCR at different template copies.

Table S1. Clinical data of patients.

Patients	Specimens	Disease (s)	HBsAg status	HBV viral load (IU/mL)
HBV 1	liver tissue (LT), serum (S), plasma (P)	HBV infection, HCC	Positive	337391

HBV 2	liver tissue (LT), serum (S), plasma (P)	HBV infection, HCC	Positive	34419
HBV 3	liver tissue (LT), serum (S), plasma (P)	HBV infection, HCC	Positive	1325
Non-HBV 1	liver tissue (LT)	HCV infection, alcoholic liver disease, HCC	Negative	Not determined
Non-HBV 2	liver tissue (LT)	Colorectal cancer, liver metastasis	Negative	Not determined
Non-HBV 3	liver tissue (LT)	Autoimmune hemolytic anemia (AIHA), rectum cancer, liver metastasis	Negative	Not determined

Table S2. Baseline characteristics of patients with overt and occult hepatitis B infection.

**Characteristics**	**Overt hepatitis B infection (n=8)**	**Occult hepatitis B infection (n=2)**	***P***
Age (Years)	61.5 ± 9.4	62.5 ± 4.9	0.8917
Female (%)	3 (37.5)	0 (0)	>0.9999
Hemoglobin (g/dL)	10.9 ± 1.6	12.2 ± 2.4	0.3807
Platelet count (10^3/μL)	128.6 ± 43.6	262.5 ± 297.7	0.1719
Total bilirubin (mg/dL)	1.079 ± 0.4	1.54 ± 1.7	0.4210
AST (IU/L)	207.4 ± 184.1	57 ± 2.8	0.3016
ALT (IU/L)	187 ± 157.7	71 ± 60.8	0.3538
ALP (IU/L)	72.75 ± 29.0	128 ± 87.7	0.1284
Albumin (mg/dL)	3.25 ± 0.6	3.25 ± 0.2	>0.9999

INR	1.226 ± 0.1	1.365 ± 0.4	0.2813
AFP (ng/mL)	21.07 ± 33.4	1.73 ± 0.6	0.4735
Cirrhosis (Yes)	6 (75)	1 (50)	>0.9999
HBsAg quantification	666.0	0.03000	0.357879
cccDNA (qPCR)	49.89	42.02	0.490068
cccDNA (CRISPR/Cas12a)	40.69	23.04	0.252773
Intrahepatic HBV DNA	145.4	51.01	0.397675
